# A modular framework for the development of targeted Covid-19 blood transcript profiling panels

**DOI:** 10.1186/s12967-020-02456-z

**Published:** 2020-07-31

**Authors:** Darawan Rinchai, Basirudeen Syed Ahamed Kabeer, Mohammed Toufiq, Zohreh Tatari-Calderone, Sara Deola, Tobias Brummaier, Mathieu Garand, Ricardo Branco, Nicole Baldwin, Mohamed Alfaki, Matthew C. Altman, Alberto Ballestrero, Matteo Bassetti, Gabriele Zoppoli, Andrea De Maria, Benjamin Tang, Davide Bedognetti, Damien Chaussabel

**Affiliations:** 1 Sidra Medicine, Doha, Qatar; 2 Shoklo Malaria Research Unit, Mahidol-Oxford Tropical Medicine Research Unit, Faculty of Tropical Medicine, Mahidol University, Mae Sot, Thailand; 3 Centre for Tropical Medicine and Global Health, Nuffield Department of Medicine, University of Oxford, Oxford, UK; 4 Swiss Tropical and Public Health Institute, Basel, Switzerland; 5 University of Basel, Basel, Switzerland; 6 Baylor Institute for Immunology Research and Baylor Research Institute, Dallas, TX USA; 7 Division of Allergy and Infectious Diseases, University of Washington, Seattle, WA USA; 8 Systems Immunology, Benaroya Research Institute, Seattle, WA USA; 9 Department of Internal Medicine, Università degli Studi di Genova, Genoa, Italy; 10 IRCCS Ospedale Policlinico San Martino, Genoa, Italy; 11 Division of Infectious and Tropical Diseases, IRCCS Ospedale Policlinico San Martino, Genoa, Italy; 12 Department of Health Sciences, University of Genoa, Genoa, Italy; 13 Nepean Clinical School, University of Sydney, Sydney, NSW Australia

**Keywords:** Blood transcriptomics, SARS-CoV-2, Covid-19, Immune monitoring

## Abstract

**Background:**

Covid-19 morbidity and mortality are associated with a dysregulated immune response. Tools are needed to enhance existing immune profiling capabilities in affected patients. Here we aimed to develop an approach to support the design of targeted blood transcriptome panels for profiling the immune response to SARS-CoV-2 infection.

**Methods:**

We designed a pool of candidates based on a pre-existing and well-characterized repertoire of blood transcriptional modules. Available Covid-19 blood transcriptome data was also used to guide this process. Further selection steps relied on expert curation. Additionally, we developed several custom web applications to support the evaluation of candidates.

**Results:**

As a proof of principle, we designed three targeted blood transcript panels, each with a different translational connotation: immunological relevance, therapeutic development relevance and SARS biology relevance.

**Conclusion:**

Altogether the work presented here may contribute to the future expansion of immune profiling capabilities via targeted profiling of blood transcript abundance in Covid-19 patients.

## Background

Covid-19 is an infectious, respiratory disease caused by a newly discovered coronavirus: SARS-CoV-2. The course of infection vary widely, with most patients presenting mild symptoms. However, about 20% of patients develop severe disease and require hospitalization [1, 2]. The interaction between innate and adaptive immunity can lead to the development of neutralizing antibodies against SARS-CoV-2 antigens that might be associated with viral clearance and protection [3]. But immune factors are also believed to play an important role in the rapid clinical deterioration observed in some Covid-19 patients [4]. There is thus a need to develop new modalities that can improve the delineation of “immune trajectories” during SARS-CoV-2 infection.

Blood transcriptome profiling involves measuring the abundance of circulating leukocyte RNA on a genome-wide scale via RNA sequencing [5]. Processing of the samples and the raw sequencing data however, is time consuming and requires access to sophisticated laboratory and computational infrastructure. Thus, the possibility of implementing this approach on large scales to ensure immediate translational potential is limited. Such unbiased omics profiling data might rather be leveraged to inform the development of more practical, scalable and targeted transcriptional profiling assays. These assays could in turn serve to significantly bolster existing immune profiling capacity.

Fixed sets of transcripts grouped based on co-expression observed in large collections of reference datasets provide a robust platform for transcriptional profiling data analyses [6]. Here we leveraged a repertoire of 382 transcriptional modules previously developed by our team [7]. The repertoire is based on a collection of reference patient cohorts encompassing 16 pathological or physiological states and 985 individual transcriptome profiles. In this proof of principle study, we used the available transcript profiling data from two separate studies to select Covid-19 relevant sets of modules [8, 9]. Next, we applied filters based on pre-specified selection criteria (e.g. immunologic relevance or therapeutic relevance). Finally, expert curation was used as the last selection step. For this we have developed custom web applications to consolidate the information necessary for the evaluation of candidates. One of these applications provides access to module-level transcript abundance profiles for available Covid-19 blood transcriptome profiling datasets. Another web interface was implemented which serves as a scaffold for the juxtaposition of such transcriptional profiling data with extensive functional annotations.

## Methods

### Datasets

Two Covid-19 blood transcriptional datasets available at the time this work was conducted were used: (1) Xiong et al. [9] obtained peripheral mononuclear cell samples obtained from one uninfected control individual and three patients with Covid-19. RNA abundance was profiled via RNAseq. The data were deposited in the Genome Sequence Archive of the Beijing Institute of Genomics, Chinese Academy of Sciences, under the accession number CRA002390. FASTQ files were downloaded from this repository. Following QC reads were aligned to reference genome GRCh38/hg19 using Hisat2 (v2.05). BAM files were converted to a raw count expression matrix using subreads (v1.6.2). Raw expression data was corrected for within lane and between lane effects using R package EDASeq (v2.12.0) and quantile normalized using preprocessCore (v1.36.0). The modular analysis was performed by using 10,617 RNA-seq genes which overlapped with transcripts from the 3rd generation module construction [7]. Details of the analysis as described below section.

(2) Ong et al. [8] collected whole blood stabilized in RNA buffer from uninfected controls and three Covid-19 patients at multiple time points. RNA abundance was profiled using a standard immunology panel from Nanostring comprising 594 transcripts. The data were deposited in the arrayexpress public repository with accession ID E-MTAB-8871. The normalized data were downloaded, and modular analysis was performed by using 403 NanoString genes which overlapped with transcripts from the 3nd generation module construction details of the analysis as described below section.

We used in addition a reference dataset generated by our group that was previously used for the construction of the 382 blood transcriptional module repertoire. This repertoire served in turn as the basis for the selection/development of targeted Covid-19 blood transcript panels described in the present article [7]. Briefly, this repertoire consists of the following cohorts of patients and respective control subjects: *S. aureus* infection (99 cases, 44 controls), sepsis (35 cases, 12 controls), tuberculosis (23 cases, 11 controls), Influenza (25 cases, 14 controls), RSV infection (70 cases, 14 controls), HIV infection (28 cases, 35 controls), systemic lupus erythematosus (55 cases, 14 controls), multiple sclerosis (34 cases, 22 controls), juvenile dermatomyositis (40 cases, 9 controls), Kawasaki disease (21 cases, 23 controls), systemic onset idiopathic arthritis (62 cases, 23 controls), COPD (19 cases, 24 controls), melanoma (22 cases, 5 controls), pregnancy (25 cases, 20 controls), liver transplant recipients (94 cases, 30 controls), and B cell deficiency (20 cases, 13 controls). All samples were run at the same facility on Illumina HumanHT-12 v3.0 Gene Expression BeadChips. The data have been deposited in NCBI Gene Expression Omnibus (GEO) with accession number GSE100150.

### Transcriptional module repertoire

The method used to construct the transcriptional module repertoire has been described elsewhere [10, 11]. The version used here is the third and last to have been developed by our group over a period of 12 years. It is the object of a separate publication (available on a pre-print server [7]).

Briefly, the approach consists of identifying sets of co-expressed transcripts in a wide range of pathological or physiological states, focusing in this case on the blood transcriptome as the biological system. We determined co-expression based on patterns of co-clustering observed for all gene pairs across the collection of 16 reference datasets listed in the previous section and that encompassed viral and bacterial infectious diseases as well as several inflammatory or autoimmune diseases, B-cell deficiency, liver transplantation, stage IV melanoma and pregnancy. Overall, this collection comprised 985 blood transcriptome profiles. A weighted, co-expression network was built with the weight of the nodes connecting a gene pair being based on the number of times co-clustering was observed for the pair among the 16 reference datasets. Thus, the weights ranged from 1 (where co-clustering occurs in one of 16 datasets) to 16 (where co-clustering occurs in all 16 datasets). Next, this network was mined using a graph theory algorithm to define subsets of densely connected gene sets that constituted our module repertoire (“Cliques” and “Paracliques”).

Overall, 382 transcriptional modules were identified, encompassing 14,168 transcripts. A supplemental file including the definition of this module repertoire along with the functional annotations is made available here (Additional file 3). To provide another level of granularity and facilitate data interpretation, a second round of clustering was performed to group the modules into “aggregates”. This process was achieved by grouping the set of 382 modules according to the patterns of transcript abundance across the 16 reference datasets that were used for module construction. This segregation resulted in the formation of 38 aggregates, each comprising between one and 42 modules.

### Module repertoire analyses

The modular analyses were performed using the core set of 14,168 transcripts forming the module repertoire. For group-level comparisons (cases vs controls), a paired t-test was performed on the log2-transformed data [Fold change (FC) cut off = 1.5; FDR cut off = 0.1]. For individual-level comparisons, each sample was compared to the mean value of the corresponding control samples (or individual sample in the case of the Xiong et al. dataset). The cut off comprised an absolute FC > 1.5 and a difference in counts > 10. The results for each module are reported as the percentage of its constitutive transcripts that increased or decreased in abundance. Group-level comparisons were performed on the reference datasets (collection of 16 datasets from Altman et al.). Individual-level comparisons were performed on both Covid-19 datasets. Because the genes comprised in a module are selected based on the co-expression observed in blood, the changes in abundance within a given module tend to be coordinated and the dominant trend is therefore selected (the greater value of the percentage increased vs. percentage decreased). Thus, the values range from -100% (all constitutive modules are decreased) to +100% (all constitutive modules are increased). A module was considered to be “responsive” when the proportion of transcripts found to be increased was > 15% (induced), or when the proportion of transcripts found to be decreased was ≤ 15% (repressed). At the aggregate-level, the percent values of the constitutive modules were averaged. Module aggregates showing little changes in Covid-19 patients were filtered out from the selection process. This was based on the proportion of modules for a given aggregate showing changes for all three subjects from the Xiong et al. dataset. The cutoff was set at 15%. In total of 17 out of the 38 module aggregates exceeded this cutoff and were thus retained for downstream analyses. They are listed in Table 1.

**Table 1:** List of Covid-19 relevant aggregates and module sets

Module aggregate	Module Set	Modules	Functional annotations
A1	A1/S1	M14.42, M15.38, M12.6, M13.27,	T cells
	A1/S2	M14.23, M15.87, M14.5, M14.49, M12.1, M14.20	Gene transcription
	A1/S3	M12.8, M15.29, M14.58, M15.51, M14.64, M16.78, M14.75, M15.82, M14.80	B cells
A2	A2/S1	M13.21, M9.1	Cytotoxic lymphocytes
	A2/S2	M14.13, M14.72, M13.13, M13.14, M14.45, M13.10, M15.91	TBD
A4	A4/S1	M16.69, M16.72, M16.50, M16.77	Antigen presentation,
A5	A5/S1	M16.95, M16.36	B cells
	A5/S2	M16.57, M16.18, M16.65, M16.111, M16.99	B cells
A7	A7/S1	M15.61	Monocytes
A8	A8/S1	M16.30	Complement
	A8/S2	M16.106	TBD
A10	A10/S1	M15.102	Prostanoids
A26	A26/S1	M12.2	Monocytes
A27	A27/S1	M13.32, M12.15, M16.92, M15.110, M16.60	Antibody producing cells
A28	A28/S1	M15.127, M8.3	Interferon response
	A28/S2	M15.64	Interferon response
	A28/S3	M15.86, M10.1, M13.17	Interferon response
A31	A31/S1	M14.81, M16.64	Platelet/Prostaglandin
	A31/S2	M14.48, M14.38, M15.58	Monocytes
A33	A33/S1	M15.104, M14.82, M14.24, M15.108	Cytokines/chemokines, Inflammation
	A33/S2	M14.19, M14.76, M14.50, M14.26, M16.101, M16.100, M16.80	Inflammation
A34	A34/S1	M14.39, M14.59, M10.3, M16.109, M8.2	Platelets, prostanoids
A35	A35/S1	M14.65, M14.28, M15.81, M16.79, M13.3, M14.7,	Monocytes, neutrophils
	A35/S2	M15.26, M12.10, M13.22, M15.109, M15.78, M13.16,	Neutrophils, inflammation
A36	A36/S1	M16.34, M16.82, M15.97, M14.51, M15.118, M16.88	Gene transcription
A37	A37/S1	M9.2, M14.53, M11.3, M12.11, M15.100 M15.74, M13.26, M13.30, M15.53,	Erythroid cells
A38	A38/S1	M10.4	Neutrophil activation
	A38/S2	M16.96, M12.9, M14.68	Erythroid cells

### Data visualization

Changes in transcript abundance reduced at the module or module aggregate-level were visualized using a custom fingerprint heatmap format. For each module, the percentage of increased transcripts is represented by a red spot and the percentage of decreased transcripts is represented by a blue spot. The fingerprint grid plots were generated using “ComplexHeatmap” [12]. A web application was developed to generate the plots and browse modules and module aggregates (https://drinchai.shinyapps.io/COVID_19_project/). A detailed description and source code will be available as part of a separate publication BioRxiv deposition on GitHub and BioRxiv (in preparation).

### Selection of transcripts for inclusion in targeted panels

#### Therapeutic relevance

Covid-19 module sets belonging to aggregates comprising module annotations relating to inflammation, monocytes, neutrophils or coagulation pathway were selected for screening (A7, A8, A26, A31, A33, A34, A35). In turn, transcripts from each of the corresponding module sets were selected on the basis of their status as a known therapeutic target of a drug for which clinical precedence exists (source: targetvalidation.org). Next, candidates were prioritized via expert curation on the basis of compatibility and a potential benefit as a Covid-19 treatment. Curators selected for this task were medical degree holders. They were provided with reports from the Open Targets website [13]. These reports included transcripts within a given module set which products were identified as being targetable by existing drugs (with tractability information indicating “with clinical precedence”; e.g. for Module M16.64 A31/S1: https://bit.ly/3dLin5P). Based on this information, their medical knowledge, and review of the relevant literature curators identified among candidates targeted by drugs those that would be most likely to be considered for treatment of Covid-19 patients. When multiple such candidates were identified a ranking was given based on feasibility and perceived potential clinical benefit. Only the top ranked candidate from each set was selected for inclusion in the panel. Module sets from aggregate A28 (interferon response) may also be of clinical relevance, as indicators of a treatment response since interferon administration has been shown to increase the activity of anti-viral drugs in Covid-19 patients [14]. The selection of candidates for aggregate A28 sets was thus based on the amplitude of the response to beta-interferon therapy measured in patients with multiple sclerosis [fold-change over pre-treatment baseline [15] & NCBI GEO accession GSE26104]. The remaining nine aggregates, which tended to associate preferentially with adaptive immune responses and for which targeting by therapies might prove detrimental, were not included in this screen. For these, representative transcripts from the default panel of immune relevant transcripts were included.

#### Relevance to Coronavirus biology

For the second panel, transcripts were primarily selected based on their relevance to SARS (Severe Acute Respiratory Syndrome) biology. As a first step, a literature profiling tool was used to identify among the SARS, MERS (Middle East Respiratory Syndrome), or Covid-19 literature articles that were associated with transcripts forming the 28 Covid-19 module sets [Literature Lab (LitLab) by Acumenta Biotech [16] and LitLab Gene Retriever application, Accumenta Biotech, Boston, MA]. Next, the potential associations were assessed by manual curation. The curators prioritized the transcripts for which the associations could be confirmed based on importance and robustness.

#### Immunological relevance

Lists of immunologically relevant genes were retrieved from Immport, the NIAID Immunology Database and Analysis Portal [17], and were used along with membership to IPA pathways (Ingenuity Pathway Analysis, QIAGEN, Germantown MD) to annotate transcripts comprising Covid-19 module sets. The curators prioritized annotated transcripts on the basis of their relevance to the functional annotations of the module set (e.g. if the main annotation for the modules for a given set is “cytotoxic cells”, markers for NK cells would be preferentially over a cytokine that is better characterized but is unrelated to cytotoxic functions). The transcript with the highest priority rank was included in the assay.

#### Housekeeping genes

A recommended set of housekeeping genes is provided in Table 2. These were selected on the basis of low variance observed across the 985 transcriptome profiles generated for our reference cohorts.

**Table 2:** List of housekeeping genes that may be suitable for blood transcript profiling applications

Housekeeping Genes	NCBI Entrez ID	Symbol	Name
Housekeeping Gene	1794	DOCK2	Dedicator of cytokinesis 2
Housekeeping Gene	1915	EEF1A1	Eukaryotic translation elongation factor 1 alpha 1
Housekeeping Gene	90268	FAM105B/OTULIN	OTU deubiquitinase with linear linkage specificity
Housekeeping Gene	2512	FTL	Ferritin light chain
Housekeeping Gene	103910	MYL12B/MRLC2	Myosin light chain 12B
Housekeeping Gene	4637	MYL6	Myosin light chain 6
Housekeeping Gene	6204	RPS10	Ribosomal protein S10
Housekeeping Gene	6230	RPS25	Ribosomal protein S25

### Annotation framework

Links to the resources described in this section and to video demonstrations are available in Table 3. Interactive presentations were created via the Prezi web application. For this we have built and expanded upon an annotation framework established as part of the characterization of our reference blood transcriptome repertoire [7]. Several bioinformatic resources were used to populate interactive presentations that served as a framework for annotation of Covid-19 relevant module sets. These resources include web applications deployed using Shiny R, which permit to plot transcript abundance patterns at the module and aggregate levels. Two of these applications were developed as part of a previous work establishing the blood transcriptome repertoire and applying it in the context of a meta-analysis of six public RSV datasets [18]. As described above, a third application was developed as part of this work and can generate profiles at the transcript, module and module-aggregate levels for the Xiong et al. and Ong et al. datasets.

**Table 3:** Resources used for annotation and interpretation

Platform/Use	Name	Source	Notes	Link	Demo video	Ref.
*Interactive presentations (Prezi)* Exploration of aggregated annotations/curation/capture interpretation of functional relevance in the context of Covid-19 disease	Covid-19 Module Sets annotation framework	In house/open	See Fig. 5 for details	A31: https://prezi.com/view/zYCSLyo0nvJTwjfJkJqb/ A28: https://prezi.com/view/7lbgGwfiNflffqQzvL14/)	Part 1: https://youtu.be/_7sNE3e5W5g Part 2: https://youtu.be/I6wHrrmbet4 Part 3: https://youtu.be/iHnM7OH_nw8	Present work
*R Shiny Web Applications/*Exploration of module-level and gene-level blood transcriptome profiling data. Design and export of custom plots to populate the annotation framework.	Covid-19 app	In house/open	Provides access to two Covid-19 blood transcript profiling datasets. More will be added as they become available.	https://drinchai.shinyapps.io/COVID_19_project/	https://youtu.be/XhQZj9mm2ME	Present work
	Gen3 app	In house/open	Provides access to 16 reference patient cohorts datasets	https://drinchai.shinyapps.io/dc_gen3_module_analysis/#	https://youtu.be/y__7xKJo5e4	Altman et al. [7]
	RSV app	In house/open	Provides access to six public RSV blood transcriptome datasets.	https://drinchai.shinyapps.io/RSV_Meta_Module_analysis/	https://youtu.be/htNSMreM8es	Rinchai et al. [18]
*Gene Expression Browser (GXB)* Interactive browsing of expression profiles for individual transcripts. Themed curated dataset collections have been created.	GXB sepsis collection	In house/open	Makes 93 curated datasets relevant to sepsis	http://sepsis.gxbsidra.org/dm3/geneBrowser/list	https://youtu.be/D1rGYfVSAoM	Toufiq et al. (in preparation), and Speake et al. [48]
			A reference dataset presenting transcript abundance profiles across purified leukocyte populations	http://sepsis.gxbsidra.org/dm3/geneBrowser/show/4000098		Linsley et al. [49]
			Reference dataset presenting the response to in vitro blood stimulations	http://sepsis.gxbsidra.org/dm3/geneBrowser/show/4000152		Obermoser et al. [22]
	GXB Acute Respiratory Infection	In house/open	34 curated datasets relevant to acute respiratory infections	http://vri1.gxbsidra.org/dm3/geneBrowser/list		Bougarn et al. [50]
			Reference dataset presenting changes in blood transcript abundance in patients with pneumonia	http://vri1.gxbsidra.org/dm3/miniURL/view/Mh		Parnell et al. [51]
*Gene Set Annotation tool.*	GSAN	Third party/open		https://gsan.labri.fr/		Allion-Benitez et al. [52]
*Pathway Analysis tool*	Ingenuity Pathway Analysis	Third party/commercial	Pathway enrichment. Used for expert curation of candidates.	https://digitalinsights.qiagen.com/products-overview/discovery-insights-portfolio/analysis-and-visualization/qiagen-ipa/		
*Accumenta Biotech LiteratureLab* Literature profiling and keyword enrichment tool	LitLab Gene Retreiver	Collaboration with Third party/commercial	Retrieves genes from a collection of literature records provided by the user	https://www.acumenta.com/generetriever		
Drug target identification	Open targets	Third party/open	Identifies drug targets among a list of gene candidates	https://www.targetvalidation.org/		
Retrieval of lists of immune-relevant genes	Immport	Third party/open	Provides access to curated gene lists	https://immport.org/shared/home		Bhattacharya et al. [17]

## Results

### Mapping Covid-19 blood transcriptome signatures against a pre-existing reference set of transcriptional modules

Changes in blood transcript abundance in response to SARS-CoV-2 infection have thus far been reported in two different studies. Different platforms, methodologies and designs were employed. Also, we first used a reference sets of signatures as a common framework in order to compare changes in transcript abundance measured in each study.

**Fig. 1:**
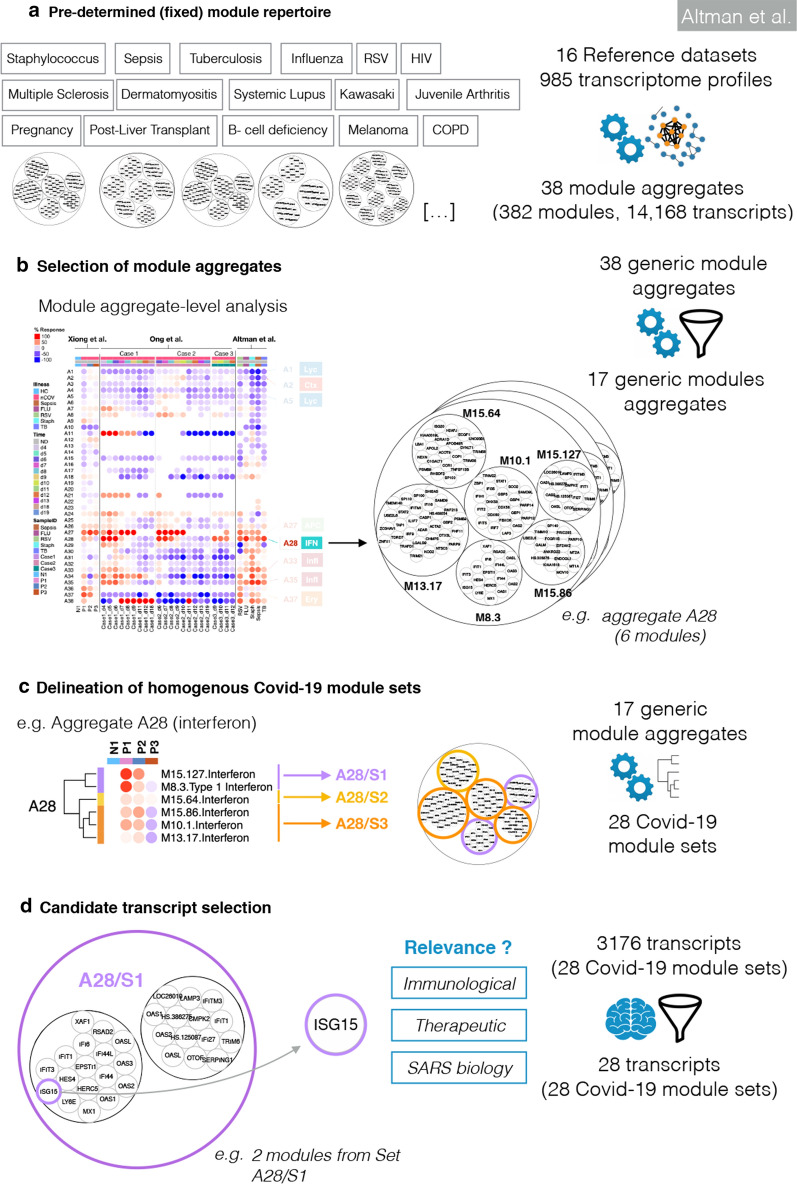
Design of targeted blood transcript panels for Covid-19 disease immune profiling. The first selection steps are data-driven (**a**–**c**). They consist in identifying co-expressed sets of transcripts to constitute “selection pools”. The last selection step is knowledge-driven (**d**). It consists in identifying transcripts among each of the selection pools which are functionally relevant for Covid-19 disease (e.g. potential therapeutic targets, molecules involved in viral entry and replication, immunological markers). **a** Pre-determined module repertoire. The process primarily relies on a generic collection of co-expressed gene sets (transcriptional modules) that were developed using an approach described in Altman et al. [7] and in the methods section. Two dimension reduction levels are built into this modular repertoire. The least reduced level has 382 variables (modules). The most reduced level has 38 variables (module aggregates, which comprise the 382 modules). **b** Selection of module aggregates. Analysis of Covid-19 patient profiles is the basis for a first down-selection step from 38 to 17 module aggregates. **c** Delineation of homogeneous Covid-19 module sets. The next step identifies within each of the 17 aggregates subsets of modules that show high degree of expression similarity across Covid-19 patients. **d** Candidate transcript selection. The last step involves expert curation and consists in identifying at least one transcript within each module set. Criteria for selection can be adapted based on needs (e.g. enrichment in candidates that are immune relevant and/or potential therapeutic targets and/or of relevance to SARS biology)

**Fig. 2:**
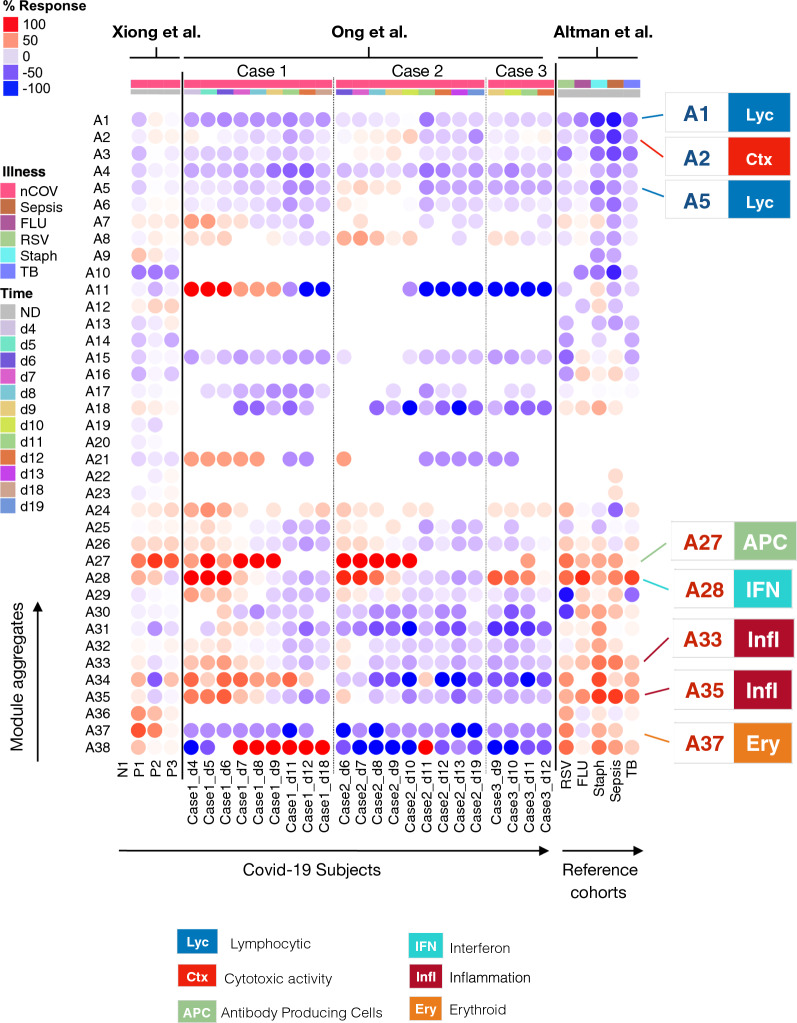
Mapping Covid-19 blood transcriptome signatures at the module aggregate level. The columns on this heatmap represent samples (Xiong et al. and Ong et al.) or patient cohorts (Altman et al.). Module aggregates (A1–A38) are arranged as rows. The colored spots represent the proportion of transcripts comprising each transcriptional module aggregate found to be differentially expressed compared to control samples. The cutoffs vary from one study to another due to differences in the design and the profiling platforms used. Thus, module aggregate response values range from 100% (all transcripts comprised in the module aggregate increased) to −100% (all decreased). The Xiong et al. dataset comprised one control and three Covid-19 patients and transcript abundance was measured by RNA-seq. The Ong et al. dataset comprised three Covid-19 cases from whom samples were collected serially, and nine uninfected controls [8]. Transcript abundance was measured using a 594 gene standard immune panel from Nanostring. Patterns are also shown for cohorts comprised in the Altman et al. dataset [7]. The colored labels (right) indicate functional associations for some of the aggregates

We employed a pre-established repertoire of 382 transcriptional modules (Fig. 1a) to map changes observed in Covid-19 patients. This module framework is described in details in “Methods” section and in a separate publication [7]. The Covid-19 datasets that we used for this were contributed by Xiong et al. [9] (one control and three subjects) and Ong et al. [8] (nine controls and three subjects profiled at multiple time points). Their data were generated using RNA-seq and Nanostring technology, respectively. The generic 594 transcript panel used by Ong et al. did not give sufficient coverage across the 382-module set. We thus mapped the transcript changes at a lower resolution, using 38 module “aggregates“(Fig. 2). These 38 aggregates encompass the entire 382 module set and constitute a more reduced version of this repertoire [see “Methods” section and [7]. In general, we saw a decrease in aggregates associated with lymphocytic compartments (aggregates A1 & A5) and an increase in aggregates associated with myeloid compartments and inflammation (aggregates A33 & A35). As expected, we also saw increases over uninfected controls for the module aggregate associated with interferon (IFN) responses (A28) and the module aggregate presumably associated with the effector humoral response (A27). We detected a wide spread of values for aggregate A11 for the Nanostring (Ong et al.) dataset. However, this aggregate comprises only one module, with only two of its transcripts measured in this Nanostring code set (the probe coverage across module aggregates is shown in Additional file 1 Fig. S1).

Despite large differences between the two studies in terms of design, range of clinical severity, technology platforms and module coverage, the combined overall changes (detected at a high-level perspective) are consistent with those observed in known acute infections, such as those caused by influenza, respiratory syncytial virus (RSV) or *S. aureus.* This consistency is evidenced by the patterns of change observed for the reference fingerprints shown alongside those of Covid-19 patients (Fig. 2).

This analysis provides a high-level mapping of changes associated with SARS-CoV-2 infection in two independent studies. It revealed a significant degree of inter-individual variability among Covid-19 patients. In one of the studies dynamic changes were also observed for the same individuals at multiple time points. Overall the analysis results show that changes in abundance of blood transcripts can be measured during the course of Covid-19 disease. It also serves to highlight the need for transcript profiling analyses to be carried out in large number of patients and at high temporal frequencies.

### Selection of aggregates and identification of coherent sets of Covid-19-relevant modules

The pre-established repertoire of 382 transcriptional modules that we have employed here covers 14,168 transcripts. It is based on co-expression patterns observed across a wide range of immune states (Fig. 1a). Also, only a fraction of the modules constituting this repertoire are expected to be of relevance for monitoring changes in transcript abundance in Covid-19 patients (as shown in Fig. 2). Thus, in the next step we selected a subset of Covid-19 relevant module aggregates (Fig. 1b). This was achieved by filtering aggregates for which seldom changes were observed among patients profiled via RNAseq by Xiong et al. (see “Methods” for details). As a result, 17 of the 38 module aggregates forming the repertoire were retained for further analysis and target selection (Table 1).

**Fig. 3:**
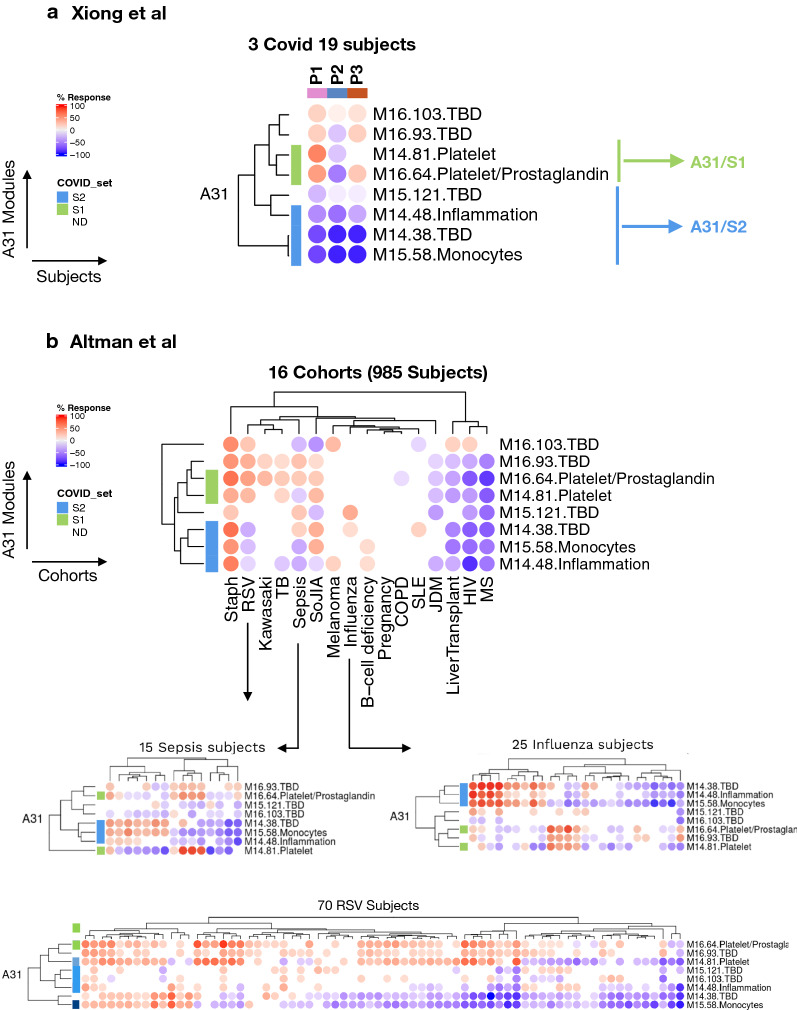
Delineation of sets of Covid-19 relevant A31 modules. **a** Transcript abundance profiles of A31 modules in Covid-19 patients. This heatmap represents the abundance levels for transcripts forming modules belonging to aggregate A31 (rows), across three Covid-19 patients (P1–P3) relative to one uninfected control subject (columns). The data are expressed as the proportion of constitutive transcripts in each module being significantly increased (red circles) or decreased (blue circles). **b** Transcript abundance profiles of A31 modules in reference disease cohorts. The top heatmap represents the abundance levels for transcripts forming modules belonging to aggregate A31 (rows), across 16 reference patient cohorts (columns). The bottom heatmaps represent the changes in abundance across the individuals comprised in two relevant patient cohorts, including pediatric patients with severe influenza or RSV infection and adult patients with sepsis

**Fig. 4:**
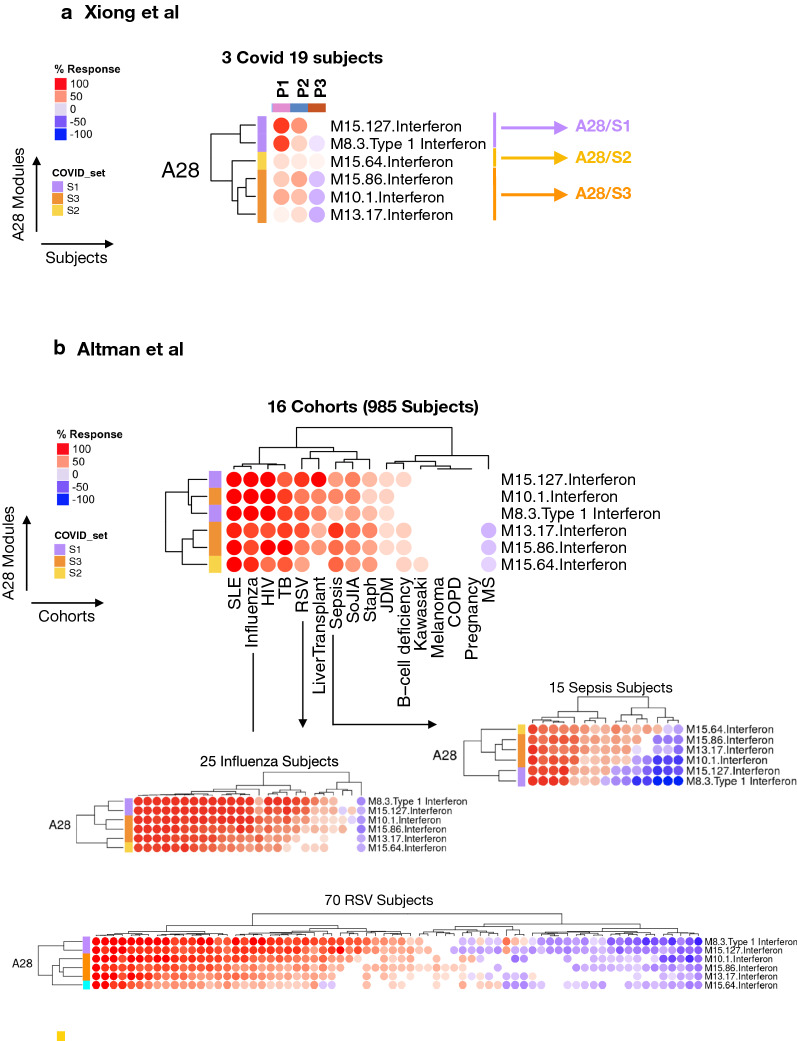
Delineation of sets of Covid-19 relevant A28 modules. **a** Transcript abundance profiles of A28 modules in Covid-19 patients. This heatmap shows the abundance levels for transcripts forming modules belonging to aggregate A28 (rows), across three Covid-19 patients (P1–P3) relative to one uninfected control subject (columns). The data are expressed as the proportion of constitutive transcripts in each module being significantly increased (red circles) or decreased (blue circles). **b** Transcript abundance profiles of A28 modules in reference disease cohorts. The top heatmap shows the abundance levels for transcripts forming modules belonging to aggregate A28 (rows), across 16 reference patient cohorts (columns). The bottom heatmaps show changes in abundance across individuals constituting the two relevant patient cohorts, including pediatric patients with severe influenza or RSV infection and adult patients with sepsis

However, patterns of changes in transcript abundance for modules comprised in a given aggregate are not always homogeneous. Thus, a further step consist in identifying sets of modules within each of the 17 aggregate that display coherent abundance patterns (Fig. 1c). To achieve this, we first mapped the changes in transcript abundance associated with Covid-19 disease using the RNAseq dataset from Xiong et al., as illustrated for A31 (Fig. 3a) and A28 (Fig. 4a). Similar plots can be generated for all other aggregates using the “COVID-19” web application (links listed in Table 3 and output provided in Additional file 2).

Next, we identified and assigned a module set ID for each the modules that formed homogeneous clusters. For example, we designated the first A28 set as A28/S1. Such module grouping is only based on patterns of transcript abundance observed in three Covid-19 patients; however, the groupings were often consistent with those observed for the much larger reference cohorts that constitute the module repertoire (Fig. 3b and Fig. 4b). A28/S1, which is formed by M8.3 and M15.127, serves as a good example of this consistency (Fig. 4b). Likewise, the segregation of the modules forming A31 based on differences observed in the three Covid-19 patients was also apparent in the reference patient cohorts (Fig. 3b). Specifically, an increase in A31/S1 modules, which accompanied a decrease in A31/S2 modules, in these three patients was also characteristic of RSV patients.

We ultimately derived 28 homogeneous Covid-19 relevant module sets from the 17 aggregates selected in the earlier step (Table 1). These sets were used as a basis for further selection.

### Design of a preliminary targeted panel emphasizing immunological relevance

In the previous step, we used available Covid-19 data to guide the selection of 28 distinct “Covid-19 relevant module sets”. In the next step, we selected the transcripts within each module set that warranted inclusion in one of three preliminary Covid-19 targeted panels. A first panel was formed using immunologic relevance as the primary criterion, a second was formed on the basis of relevance to coronavirus biology, a third was constituted on the basis of relevance to therapy.

For the first panel we matched transcripts comprised in each module set to a list of canonical immune genes (see “Methods” for details). Expert curation also involved accessing transcript profiling data from the reference datasets, indicating for instance leukocyte restriction or patterns of response to a wide range of immune stimuli in vitro. We describe our approach for module and gene annotation in more detail below and provide access to our resources to support expert curation (Table 3).

For our illustrative case, we selected one representative transcript per module set to produce a panel comprised of 28 representative transcripts (Table 4). Examples of signatures surveyed by such a panel include: (1) ISG15 in A28/S1 (interferon responses), which encodes for a member of the ubiquitin family. ISG15 plays a central role in the host defense to viral infections [19]. (2) GATA1 in A37/S1 (erythroid cells), which encodes for a master regulator of erythropoiesis [20]. It is associated with a module signature (A37) that we recently reported as being associated with immunosuppressive states, such as late stage cancer and maintenance immunosuppressive therapy in solid organ transplant recipients [18]. In the same report we also found an association between this signature and heightened severity in patients with RSV infection and established a putative link with a population of immunosuppressive circulating erythroid cells [21]. (3) CD38 in A27/S1 (cell cycle), which encodes for the CD38 molecule expressed on different circulating leukocyte populations. In whole blood we find the abundance of its transcript correlate with that of IGJ, TNFRSF17 (BCMA), TXNDC5 (M12.15). Such a signature was previously found to be increased in response to vaccination at day 7 post administration, to correlate with the prevalence of antibody producing cells, and the development of antibody titers at day 28 [22]. (4) TLR8 in A35/S1 (inflammation), encodes toll-like receptor 8. Expression of transcripts comprising this aggregate is generally restricted to neutrophils and robustly increased during sepsis (e.g. as we have described in detail earlier for ACSL1, another transcript belonging to this aggregate [23]). (5) GZMB in A2/S1 (Cytotoxic cells) encodes Granzyme B, a serine protease known to play a role in immune-mediated cytotoxicity. Other transcripts forming this panel are listed in Table 4.

**Table 4:** Preliminary targeted panel—immunology relevance focus

Module set	Module ID	NCBI Entrez ID	Symbol	Name	Module set functional annotation
A1/S1	M15.38	916	CD3E	CD3e molecule	T cells
A1/S2	M14.49	974	CD79B	CD79b molecule	Gene transcription
A1/S3	M14.80	3122	HLA-DRA	Major histocompatibility complex, class II, DR alpha	B cells
A2/S1	M9.1	3002	GZMB	Granzyme B	Cytotoxic lymphocytes
A2/S2	M13.13	4282	MIF	Macrophage migration inhibitory factor	TBD
A4/S1	M16.77	3811	KIR3DL1	Killer cell immunoglobulin like receptor, three Ig domains and long cytoplasmic tail 1	Antigen presentation
A5/S1	M16.95	972	CD74	CD74 molecule	B cells
A5/S2	M16.111	27242	TNFRSF21	TNF receptor superfamily member 21	B cells
A7/S1	M15.61	23166	STAB 1	Stabilin 1	Monocytes
A8/S1	M16.30	3600	IL15	Interleukin 15	Complement
A8/S2	M16.106	57823	SLAMF7	SLAM family member 7	TBD
A10/S1	M15.102	246	ALOX15	Arachidonate 15-lipoxygenase	Prostanoids
A26/S1	M12.2	942	CD86	CD86 molecule	Monocytes
A27/S1	M12.15	608	CD38	CD38 molecule	Cell cycle
A28/S1	M8.3	9636	ISG15	ISG15 ubiquitin like modifier	Interferon response
A28/S2	M15.64	10475	TRIM38	Tripartite motif containing 38	Interferon response
A28/S3	M10.1	115362	GBP5	Guanylate binding protein 5	Interferon response
A31/S1	M16.64	1950	EGF	Epidermal growth factor	Platelet/prostaglandin
A31/S2	M15.58	2214	FCGR3A	Fc fragment of IgG receptor IIIa	Monocytes
A33/S1	M14.24	91	ACVR1B	Activin A receptor type 1B	Cytokines/chemokines, Inflammation
A33/S2	M14.19	23765	IL17RA	Interleukin 17 receptor A	Inflammation
A34/S1	M8.2	3674	ITGA2B	Integrin subunit alpha 2b	Platelets, prostanoids
A35/S1	M13.3	1241	LTB4R	Leukotriene B4 receptor	Inflammation
A35/S2	M12.10	51311	TLR8	Toll like receptor 8	Neutrophils, inflammation
A36/S1	M16.34	2993	GYPA	Glycophorin A (MNS blood group)	Gene transcription
A37/S1	M11.3	2623	GATA1	GATA binding protein 1	Erythroid cells
A38/S1	M10.4	4057	LTF	Lactotransferrin	Neutrophil activation
A38/S2	M16.96	56729	RETN	Resistin	Erythroid cells

Even with the limited amount of data available to guide the selection in the previous steps, it is reasonable to assume that such a panel (while not optimal) would already provide valid information for Covid-19 immune profiling. Additional Covid-19 blood transcriptome data that will become available in the coming weeks will allow us to refine the overall selection process.

### Design of a preliminary targeted panel emphasizing therapeutic relevance

**Fig. 5:**
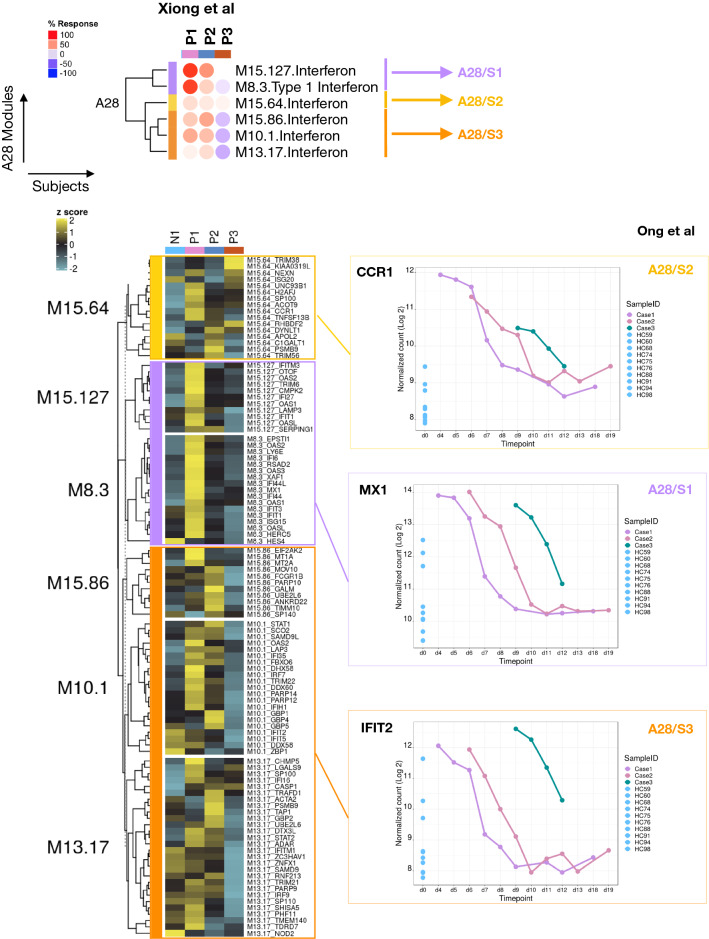
Changes in abundance of transcripts comprising aggregate A28 in response to SARS-CoV-2 infection. The heatmaps display the changes in transcript abundance in three Covid-19 patients comprising the Xiong et al. RNA-seq transcriptome dataset. The top heatmap summarizes the module-level values for the six modules forming aggregate A28. The color code indicates membership to one of the three Covid-19 module sets that were defined earlier. The bottom heatmap shows patterns of abundance for the same six modules, but at the individual gene level. The line graphs on the right show changes in abundance for three transcripts from the “therapeutic relevance panel” in three Covid-19 patients profiled by Ong et al. using a generic Nanostring immune set comprising 594 transcripts

A different translational connotation was given for this second panel. Here, we based the selection on the same collection of 28 module sets. However, this time, whenever possible, we included transcripts that could have value as targets for the treatment of Covid-19 patients. An initial screen identified 82 transcripts encoding molecules that are known targets for existing drugs (see “Methods”). We further prioritized these candidates based on an expert’s evaluation of the compatibility of use of the drugs for treating Covid-19 patients. As an exception, module sets belonging to A28 (interferon response) were selected based on their suitability as markers of a response to interferon therapy (as described in “Methods” and illustrated in Fig. 5). Sets for which no targets of clinical relevance were identified (16/28) were instead represented in the panel by immunologically-relevant transcripts (defined earlier). Indeed, while it is possible to customize panels according to preference or needs, it would be optimal for any such custom targeted panel to maintain coverage across the entire breadth of Covid-19 signatures (i.e. the 28 homogenous Covid-19 module sets).

We ultimately identified a preliminary set of 12 targets through this high stringency selection process (Table 5). Developing effective immune modulation therapies in critical care settings has proven challenging [24]. Current efforts in the context of Covid-19 disease particularly aim at controlling runaway systemic immune responses or so called “cytokine storms” that have been associated with organ damage and clinical worsening. Targets of interest identified among our gene set include: (1) IL6R in A35/S2 (inflammation), encoding the Interleukin-6 Receptor, which is a target for the biologic drug Tocilizumab. Several studies have tested this antagonist in open label single arm trials in Covid-19 patients with the intent of blocking the cytokine storm associated with severe Covid-19 infection [25, 26]. (2) CCR2 in A26/1 (monocytes), encoding the chemokine (C–C motif) receptor 2, is targeted along with CCR5 by the drug Cenicriviroc. This drug exerts potent anti-inflammatory activity [27]. (3) TBXA2R in A31/1 (platelets), encoding the Thromboxane receptor, is targeted by several drugs with anti-platelet aggregation properties [28]. (4) PDE8A in A33/S1 (inflammation), encoding Phosphodiesterase 8A, is targeted by Pentoxifylline, a non-selective phosphodiesterase inhibitor that increases perfusion and may reduce risk of acute kidney injury and attenuates LPS-induced inflammation [29]. (5) NQO1 in A8/S1 (Complement), encoding NAD(P)H quinone dehydrogenase 1. The NQO1 antagonist Vatiquinone (EPI-743) has been found to inhibit ferroptosis [30], a process associated with tissue injury [31], including in sepsis [32]. A complete list is provided in Table 5.

**Table 5:** Preliminary targeted panel—therapeutic relevance focus

Module set	Module ID	NCBI Entrez ID	Symbol	Name	Relevance	Notes
A1/S1	M15.38	916	CD3E	CD3e molecule	Immunological	Not suitable for targeting (adaptive immunity)
A1/S2	M14.49	974	CD79B	CD79b molecule	Immunological	Not suitable for targeting (adaptive immunity)
A1/S3	M14.80	3122	HLA-DRA	Major histocompatibility complex, class II, DR alpha	Immunological	Not suitable for targeting (adaptive immunity)
A2/S1	M9.1	3002	GZMB	Granzyme B	Immunological	Not suitable for targeting (adaptive immunity)
A2/S2	M13.13	4282	MIF	Macrophage migration inhibitory factor	Immunological	Not suitable for targeting (adaptive immunity -presumed)
A4/S1	M16.77	3811	KIR3DL1	Killer cell immunoglobulin like receptor, three Ig domains and long cytoplasmic tail 1	Immunological	Not suitable for targeting (adaptive immunity)
A5/S1	M16.95	972	CD74	CD74 molecule	Immunological	Not suitable for targeting (adaptive immunity)
A5/S2	M16.111	27242	TNFRSF21	TNF receptor superfamily member 21	Immunological	Not suitable for targeting (adaptive immunity)
A7/S1	M15.61	23166	STAB 1	Stabilin 1	Immunological	No suitable candidates identified
A8/S1	M16.30	1728	NQO1	NAD(P)H quinone dehydrogenase 1	Therapeutic	Vatiquinone (EPI-743) has been found to inhibit ferroptosis [30], a process associated with tissue injury [31], including in sepsis [32]
A8/S2	M16.106	57823	SLAMF7	SLAM family member 7	Immunological	No suitable candidates identified
A10/S1	M15.102	246	ALOX15	Arachidonate 15-lipoxygenase	Immunological	No suitable candidates identified
A26/S1	M12.2	729230	CCR2	C–C motif chemokine receptor 2	Therapeutic	Anti-inflammatory properties have been attributed to the CCR2/CCR5 blocker Cenicriviroc [53]
A27/S1	M12.15	608	TNFRSF17	TNF receptor superfamily member 17	Immunological	Not suitable for targeting (adaptive immunity)
A28/S1	M8.3	4599	MX1	MX dynamin like GTPase 1	Therapeutic	Inducible by Interferon-beta treatment
A28/S2	M15.64	1230	CCR1	C–C motif chemokine receptor 1	Therapeutic	Inducible by Interferon-beta treatment
A28/S3	M10.1	3433	IFIT2	Interferon induced protein with tetratricopeptide repeats 2	Therapeutic	Inducible by Interferon-beta treatment
A31/S1	M16.64	6915	TBXA2R	Thromboxane A2 receptor	Therapeutic	Thromboxane A2 synthase inhibitors have antiplatelet aggregation activities and anti-inflammatory activities (drugs include: Defibrotide/Seratrodast, Ozagrel)
A31/S2	M15.58	5743	PTGS2	Prostaglandin-endoperoxide synthase 2	Therapeutic	PTGS2 encodes COX-2. Several specific inhibitors are available which possess anti-inflammatory properties (e.g. celecoxib, rofecoxib, valdecoxib)
A33/S1	M15.104	5151	PDE8A	Phosphodiesterase 8A	Therapeutic	PDE8A, is targeted by Pentoxifylline, a non-selective phosphodiesterase inhibitor that increases perfusion and may reduce risk of acute kidney injury and attenuates LPS-induced inflammation
A33/S2	M14.19	23765	IL17RA	Interleukin 17 receptor A	Therapeutic	Brodalumab may be beneficial in reducing the viral illness exacerbation. But current recommendation is discontinuation of use in COVID 19
A34/S1	M16.109	5742	PTGS1	Prostaglandin-endoperoxide synthase 1	Therapeutic	Encodes for Cox-1. COX inhibitors including Aspirin, Indomethacin, Naproxen have direct antiviral properties as well as anti-inflammatory and antithrombotic properties
A35/S1	M14.7	5293	JAK2	Janus kinase 2	Therapeutic	A targeted for the biologic drug Ruxolitinib. Ruxolitinib acts on cellular components of both innate and adaptive immunity inhibiting downstream cellular signaling pathways of major inflammatory mediators (e.g., IFN-alpha via JAK2, and IL-2 and IL-6 via JAK1)
A35/S2	M15.109	3570	IL6R	Interleukin 6 receptor	Therapeutic	IL6R is a target for the biologic drug Tocilizumab. Several studies have tested this antagonist in open label single arm trials in Covid-19 patients with the intent of blocking the cytokine storm associated with Covid-19 disease [15, 16]
A36/S1	M16.34	2993	GYPA	Glycophorin A (MNS blood group)	Immunological	Not suitable for targeting (erythropoiesis)
A37/S1	M11.3	2623	GATA1	GATA binding protein 1	Immunological	Not suitable for targeting (erythropoiesis)
A38/S1	M10.4	4057	LTF	Lactotransferrin	Immunological	No suitable candidates identified
A38/S2	M16.96	56729	RETN	Resistin	Immunological	Not suitable for targeting (erythropoiesis)

The fact that this transcript panel and the previous survey the same pre-defined 28 homogenous Covid-19 relevant module sets should make them largely synonymous (since modules are formed on the basis of co-expression). Nevertheless, this second panel may be more relevant for investigators interested in investigating new therapeutic approaches or measuring responses to treatment.

### Design of a preliminary targeted panel of blood transcripts of relevance for SARS-CoV-2 biology

For the third panel designed in this proof of principle, we primarily selected transcripts based on their relevance to SARS biology. As a first step, we used a literature profiling tool to identify SARS, MERS, or Covid-19 literature articles that were associated with transcripts forming the 28 Covid-19 module sets. Next, the potential associations were subjected to expert curation (see “Methods”). Once again, to keep redundancies to a minimum, we only included one candidate per set in this panel (Table 6). Notable examples include: (1) LTF in A38/S1 (Neutrophil activation), encoding Lactotransferrin, that is known to block the binding of the SARS-CoV spike protein to host cells, thus exerting an inhibitory function at the viral attachment stage [33]. (2) FURIN in A37/S1 (Erythroid cells), encodes a proprotein convertase that preactivates SARS-CoV-2, thus reducing its dependence on target cell proteases for entry [34]. (3) EGR1 in A7/S1 (Monocytes), encoding Early Growth Response 1, which upon induction by SARS Coronavirus Papain-Like Protease mediates up-Regulation of TGF-β1 [35]. (4) STAT1 in A28/S3 (Interferon response), encoding a transcription factor known to play an important role in the induction of antiviral effector responses. It was reported that SARS ORF6 antagonizes STAT1 function by preventing its translocation to the nucleus and acts as an interferon antagonist in the context of SARS-CoV infection [36].

**Table 6:** Preliminary targeted panel—SARS biology relevance focus

Module set	Module ID	NCBI Entrez ID	Symbol	Name	Relevance	Notes
A1/S1	M15.38	916	CD3E	CD3e molecule	Immunological	
A1/S2	M12.1	60489	APOBEC3G	apolipoprotein B mRNA editing enzyme catalytic subunit 3G	CoV Biology	APOBEC3G associates with SARS viral structural proteins [54], with a possible role in restriction of RNA virus replication [55]
A1/S3	M14.64	51284	TLR7	Toll like receptor 7	CoV Biology	TLR7 Signaling Pathway is inhibited by SARS Coronavirus Papain-Like Protease [35]
A2/S1	M13.21	3458	IFNG	Interferon gamma	CoV Biology	Interferon-gamma and interleukin-4 Downregulate Expression of the SARS Coronavirus Receptor ACE2 [56]
A2/S2	M13.10	25	ABL1	ABL proto-oncogene 1, non-receptor tyrosine kinase	CoV Biology	Abl Kinase inhibitors block SARS-Cov fusion [57]
A4/S1	M16.77	3811	KIR3DL1	killer cell immunoglobulin like receptor, three Ig domains and long cytoplasmic tail 1	Immunological	The inhibitory KIR3DL1 is a strong ligand for HLA Bw4, C1 and C2 groups. High expression of this inhibitory KIR was associated with slower disease progress to AIDS and better HIV viral load control [58]
A5/S1	M16.65	4092	SMAD7	SMAD family member 7	CoV Biology	MERS Coronavirus Induces Apoptosis in Kidney and Lung by Upregulating Smad7 and FGF2 [59]
A5/S2	M16.111	27242	TNFRSF21	TNF receptor superfamily member 21	Immunological	
A7/S1	M15.61	1958	EGR1	Early growth response 1	CoV Biology	SARS Coronavirus Papain-Like Protease Induces Egr-1-dependent Up-Regulation of TGF-β1 [60]
A8/S1	M16.30	857	CAV1	Caveolin 1	CoV Biology	Severe Acute Respiratory Syndrome Coronavirus Orf3a Protein Interacts with Caveolin [61]
A8/S2	M16.106	57823	SLAMF7	SLAM family member 7	Immunological	
A10/S1	M15.102	246	ALOX15	arachidonate 15-lipoxygenase	Immunological	
A26/S1	M12.2	942	CD86	CD86 molecule	Immunological	
A27/S1	M12.15	608	TNFRSF17	TNF receptor superfamily member 17	Immunological	
A28/S1	M8.3	9636	ISG15	ISG15 ubiquitin like modifier	CoV Biology	SARS-CoV PLpro exhibits ISG15 precursor processing activities [62]
A28/S2	M15.64	1230	CCR1	C–C motif chemokine receptor 1	CoV Biology	MLN-3897, a CCR1 antagonist inhibits replication of SARS-CoV-2 replication [63]
A28/S3	M10.1	6772	STAT1	Signal transducer and activator of transcription 1	CoV Biology	SARS ORF6 Antagonizes STAT1 Function [36]
A31/S1	M16.64	1950	EGF	Epidermal growth factor	Immunological	
A31/S2	M15.58	5743	PTGS2	Prostaglandin-endoperoxide synthase 2	CoV Biology	Encodes COX2, which expression is stimulated by SARS Spike protein [64]
A33/S1	M14.24	114548	NLRP3	NLR family pyrin domain containing 3	CoV Biology	Multiple SARS-Coronavirus protein have been reported to activates NLRP3 inflammasomes [65, 66]
A33/S2	M14.19	23765	IL17RA	Interleukin 17 receptor A	Immunological	
A34/S1	M8.2	3674	ITGA2B	Integrin subunit alpha 2b	Immunological	
A35/S1	M13.3	1241	LTB4R	Leukotriene B4 receptor	Immunological	
A35/S2	M15.78	290	ANPEP	alanyl aminopeptidase, membrane	CoV Biology	A potential receptor for human CoVs [67]
A36/S1	M16.88	6352	CCL5	C–C motif chemokine ligand 5	CoV Biology	CCL5/RANTES is associated with the replication of SARS in THP-1 Cells [68]
A37/S1	M13.26	5045	FURIN	Furin, paired basic amino acid cleaving enzyme	CoV Biology	Furin cleavage of the SARS coronavirus spike glycoprotein enhances cell–cell fusion [69]
A38/S1	M10.4	4057	LTF	Lactotransferrin	CoV Biology	Lactotransferrin blocks the binding of the SARS-CoV spike protein to host cells, thus exerting an inhibitory function at the viral attachment stage [33]
A38/S2	M12.9	1508	CTSB	Cathepsin B	CoV Biology	Activation of SARS- and MERS-coronavirus is mediated cathepsin L (CTSL) and cathepsin B (CTSB) [70]

This screen identified several molecules that may be of importance for SARS-CoV-2 entry and replication. A complete list is provided in Table 6. It is expected that this knowledge will evolve rapidly over time and periodic updates may be necessary. And, as for the previous two panels, investigators may also have an interest in including more than one candidate per module set. This of course would also be feasible, although at the expense of course of parsimony.

### Development of an annotation framework in support of signatures curation efforts

A vast amount of information is available to support the work of expert curators who are responsible for finalizing the selection of candidates. This process often requires accessing a number of different resources (e.g. those listed in Table 3). Here we have built upon earlier efforts to aggregate this information in a manner that makes it seamlessly accessible by the curators.

As proof of principle, we created dedicated, interactive presentations in Prezi for module aggregates A28 (https://prezi.com/view/7lbgGwfiNflffqQzvL14/) and A31 (https://prezi.com/view/zYCSLyo0nvJTwjfJkJqb/). These presentations are intended, on the one hand, to aggregate contextual information that can serve as a basis for data interpretation. On the other hand, they are intended to capture the results of the interpretative efforts of expert curators.

**Fig. 6:**
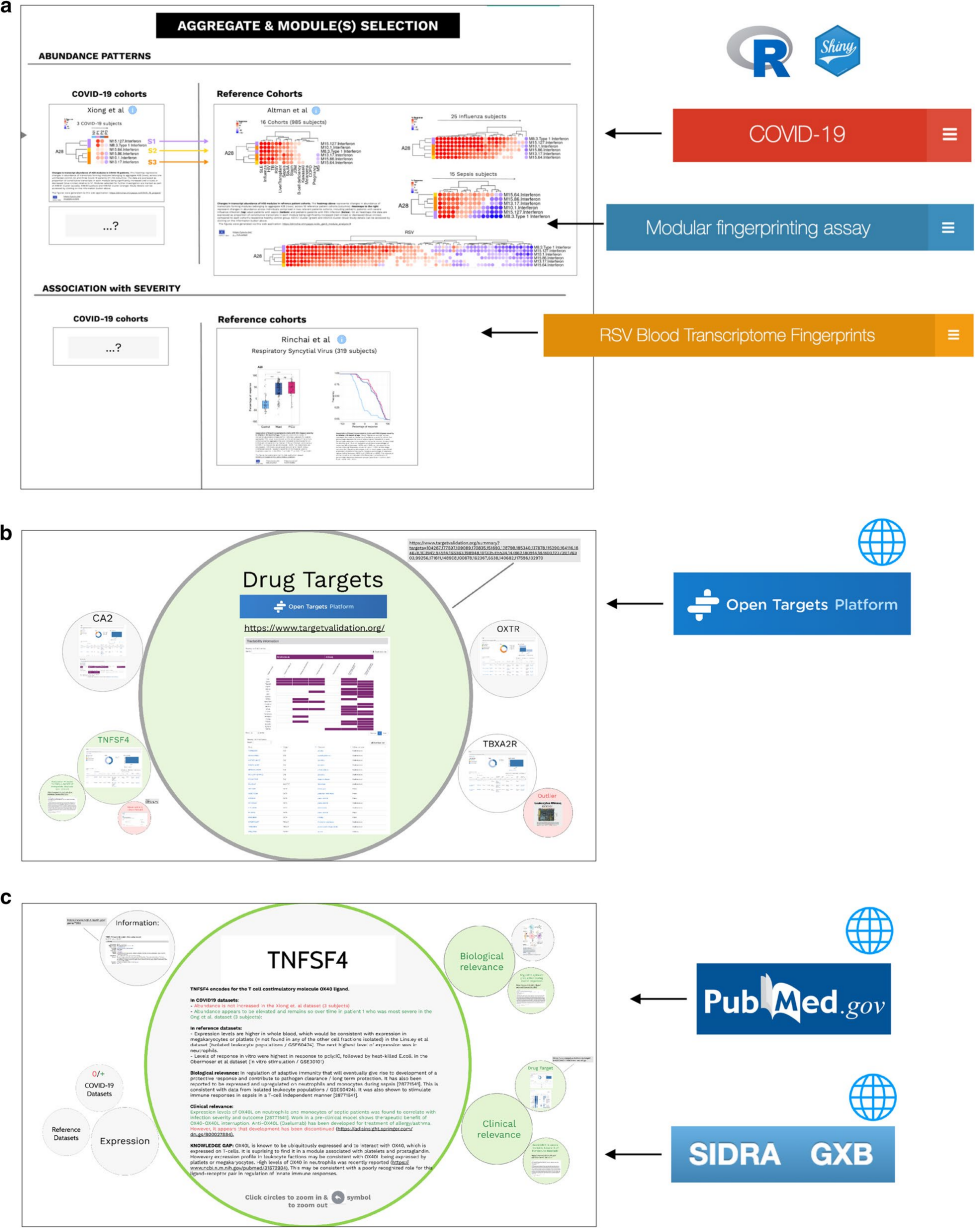
High resolution annotation framework supporting the curation and interpretation of Covid-19 module sets. This series of screenshots shows the content of the interactive presentations that have been established to provide curators with access to detailed annotations regarding modules forming a given aggregate, its constitutive modules and targets that have been selected for inclusion in transcript panels. Links to interactive presentations and resources mentioned below are available in Table 3. **a** Module aggregate-level information. This section displays patterns of transcript abundance across the modules forming a given aggregate, as well as the degree of association of this aggregate with the severity of RSV disease. Plots used to populate this section were generated using three web applications, including one that was developed in support of this work that compiles the Covid-19 blood transcriptional data available to date. The other two applications were developed as part of a previous study to generate plots for the reference disease cohorts and RSV severity association plots [47]. **b** Module-level information. This section includes, for a given module, reports from functional profiling tools as well as patterns of transcript abundance across the genes forming the module. Drug targeting profiles were added to provide another level of information. **c** Gene-centric information. The information includes curated pathways from the literature, articles and reports from public resources. Gene-centric transcriptional profiles that are available via gene expression browsing applications deployed by our group are also captured and used for context (GXB). A synthesis of the information gathered by expert curation and potential relevance to SARS-Cov-2 infection can also be captured and presented here

The interactive presentations are organized in sections, each showing aggregated information from a different level: module-sets, modules and transcripts (Fig. 6). The information derived from multiple online sources, including both third party applications and custom applications developed by our team (Table 3). Among those is a web application developed specifically for this work, which was used to generate the Covid-19 plots from Ong et al. and Xiong et al. (Figure 6a). The interactive presentation itself permits to zoom in and out, determine spatial relationships and interactively browse the very large compendium of analysis reports and heatmaps generated as part of these annotation efforts (Fig. 6b). The last section that contains transcript-centric information, is also the area where interpretations from individual curators can be aggregated (Fig. 6c).

We have annotated and interpreted some of the transcripts included in A31/S1 in such a manner: (1) OXTR, which encodes for the Oxytocin receptor through which anti-inflammatory and wound healing properties of Oxytocin are mediated [37]. Among our reference cohort datasets, OXTR is most highly increased in patients with *S. aureus* infection or active pulmonary tuberculosis [7]. (2) CD9, which encodes a member of the tetraspanin family, facilitates the condensation of receptors and proteases activating MERS-CoV and promoting its rapid and efficient entry into host cells [38]. (3) TNFSF4, which encodes for OX40L and is a member of the TNF superfamily. Although OX40L is best known as a T-cell co-stimulatory molecule, reports have also shown that it is present on the neutrophil surface [39]. Furthermore, OX40L blockade improved outcomes of sepsis in an animal model.

Our interpretation efforts have been limited thus far by expediency. Certainly, interpretation will be the object of future, more targeted efforts. In the meantime, this annotation framework supports the selection of candidates forming the panels presented here. It may also serve as a resource for investigators who wish to design custom panels of their own.

## Discussion

Early reports point to profound immunological changes occurring during the course of SARS-CoV-2 infection [40, 41]. In particular, patterns of immune dysfunction have been associated with rapid worsening of symptoms and the onset of severe respiratory failure [42]. However, disease outcomes remain highly heterogeneous and factors contributing to clinical deterioration are poorly understood. Among other modalities, means to enhance immune monitoring capabilities in cohorts of Covid-19 patients are needed. Here we designed an approach to select and curate targeted blood transcript panels relevant to Covid-19 disease.

The sparsity of the Covid-19 blood transcriptome data available to guide the selection process described in this paper was an obvious limitation. Xiong et al. dataset comprised profiles of only three Covid-19 subjects and one uninfected control subject. More transcriptome profiling data will be generated and become available in the coming weeks and months, including from our group. This will permit to re-iterate the selection process and refine the design of the preliminary versions of the three Covid-19 transcript panels being presented here. Additional Covid-19 data would likely permit to adjust the filtering of module aggregates (Fig. 1b) and improve the delineation of Covid-19 module sets (Fig. 1c). However, the generic module repertoire that serves as the main framework for candidate selection would remain unchanged (Fig. 1a). Likewise, knowledge-driven prioritization of candidates based on relevance to therapy, immunology or SARS biology is by definition independent from Covid-19 profiling data availability (Fig. 1d). Therefore, while changes to the preliminary panels presented in Tables 3, 5 and 6 resulting from additional Covid-19 profiling data becoming available are to be expected those may not prove to be extensive.

The targeted panel design approach that we are presenting is also partly knowledge-driven. Indeed, we have relied on expert knowledge for the identification of transcripts coding for molecules with biological significance or therapeutic relevance, specifically in the context of Covid-19 disease (Fig. 1d). While it was possible to enroll the help of several curators to work in parallel on this task the amount of time allotted was limited by the need for expediency. Curation of candidates is therefore another area that will be worth revisiting over the coming weeks and months as targeted blood transcript panels are further refined. It is also an effort that the platform we have developed for the aggregation of vast amounts of information from various sources would help support. This will become especially important now that an increasing number of bioinformatics tools and resources are being made available by the scientific community for tackling the current health crisis (e.g. more specifically for drug target identification and repurposing: [43, 44]).

In the illustrative use cases that we are providing non-synonymous targeted panels were formed by selecting only one representative transcript from each of the 28 homogenous Covid-19 module sets. It is nevertheless possible to devise custom selection strategies where more than one transcript is retained from each set. Our own implementation of a preliminary targeted Covid-19 blood transcriptional assay will be based on the Fluidigm Biomark high throughput PCR platform. The panel will comprise 96 targets in order to comply with the format of Fluidigm’s integrated fluidics circuits (96 samples × 96 reactions). These will include all transcripts listed in Tables 4, 5 and 6 (53 unique transcripts) which will be complemented by 35 additional candidates which received priority ranking from our expert curators and 8 housekeeping genes. While the number of candidates to be selected within a given module set remains flexible our recommendation when designing such a targeted panel would be for all 28 module sets to be covered by at least one transcript. Additional file 3 includes the list of the genes included in the modules forming the 28 Covid-19 module sets. Other medium-throughput technology platforms, such as the Nanostring nCounter System or ThermoFisher Openarray, would also be appropriate for implementing custom profiling assays with the number of targets comprising the preliminary panels presented here (or a combination thereof). Downsizing panels to comprise ± 10 key markers might serve as a basis for implementation on more ubiquitous real-time PCR platforms.

Monitoring of “immune trajectories” associated with response to SARS-CoV-2 infection and clinical deterioration of Covid-19 patients is one possible application for such a targeted assay. Another would be the measurement of responses to therapy (as part of standard of care or a trial). The immune profiling of asymptomatic or pre-symptomatic patients (e.g. quarantined) would be another setting where implementation of such an assay could prove useful. For this, it would for instance be possible to use protocols that we have previously developed for home-based, self-sampling and blood RNA stabilization [45, 46].

## Conclusions

Overall, this work lays the ground for a framework designed to support the development of interpretable targeted panels for profiling immune responses to SARS-CoV-2 infection. It consists, on one hand, in an analytic pipeline for data-driven selection of targets. And, on the other hand, in an information aggregation platform supporting the work of expert curators. The preliminary blood transcript panels presented here will be leveraged for a first round of implementation of a targeted Covid-19 immune profiling assay.

## Supplementary information

**Additional file 1: Figure S1.** Coverage of the pre-established 38 transcriptional module aggregate repertoire by the Nanostring immunology panel 2. The bar graphs show the distribution of the 579 transcript constituting the standard Nanostring immunology panel used by Ong et al. across the 38 module aggregates forming this repertoire. The Venn diagram shows the degree of overlap between the Nanostring panel and the transcripts forming this modular repertoire.

**Additional file 2.** Delineation of Covid-19 relevant modules sets in all 17 aggregates retained in the first step of the selection process.

**Additional file 3.** Composition and annotation of the 382 module repertoire employed as a framework for the selection of targeted Covid-19 blood transcriptional panels. Membership to the 28 Covid-19 module sets is indicated in column D.

## Data Availability

The datasets generated during and/or analysed during the current study are available: in the ArrayExpress repository, under the accession number E-MTAB-8871, https://www.ebi.ac.uk/arrayexpress/experiments/E-MTAB-8871/ in the Genome Sequence Archive of the Beijing Institute of Genomics, Chinese Academy of Sciences, under the accession number CRA002390, https://bigd.big.ac.cn/gsa/browse/CRA002390 In the NCBI GEO database, under the accession numbers GSE26104, https://www.ncbi.nlm.nih.gov/geo/query/acc.cgi?acc=GSE26104 GSE100150, https://www.ncbi.nlm.nih.gov/geo/query/acc.cgi?acc=GSE100150.

## Abbreviations

SARS: Severe Acute Respiratory Syndrome
MERS: Middle East Respiratory Syndrome
RSV: Respiratory Syncytial Virus
GEO: Gene Expression Omnibus
