# Supplementary figures and images for: A modular framework for the development of targeted Covid-19 blood transcript profiling panels
Source: J Transl Med. 2020 Jul 31;18:291. doi: 10.1186/s12967-020-02456-z (PMC7393249; doi:10.1186/s12967-020-02456-z)

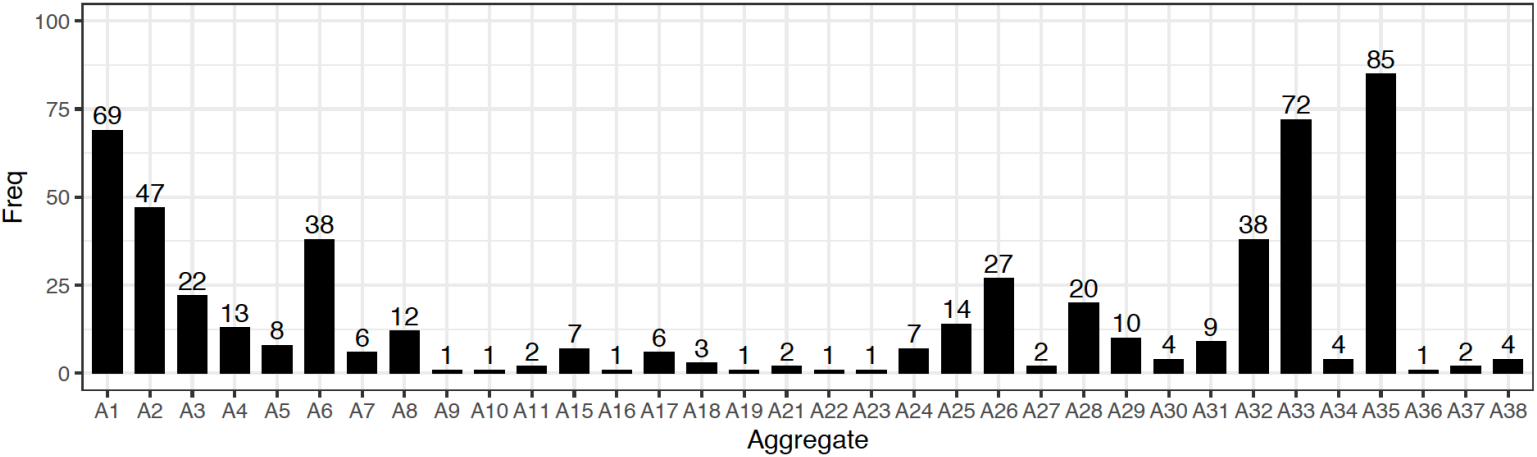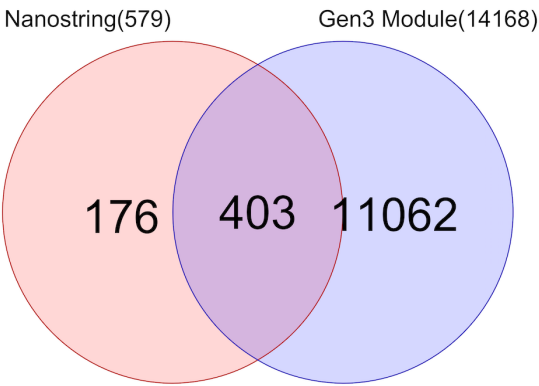

Supplementary Figure 1

Supplement: Supplementary file 1 — Additional file 1: Figure S1. Coverage of the pre-established 38 transcriptional module aggregate repertoire by the Nanostring immunology panel 2. The bar graphs show the distribution of the 579 transcript constituting the standard Nanostring immunology panel used by Ong et al. across the 38 module aggregates forming this repertoire. The Venn diagram shows the degree of overlap between the Nanostring panel and the transcripts forming this modular repertoire. [file 12967_2020_2456_MOESM1_ESM.pdf]
